# Numerical modelling approach for estimation of a yield zone in the face of a deep longwall panel

**DOI:** 10.1038/s41598-023-47683-8

**Published:** 2023-11-27

**Authors:** Sreenivasa Rao Islavath

**Affiliations:** Department of Mining Engineering, Indian Institute of Technology Kharagpur, Kharagpur, West Bengal 721302 India

**Keywords:** Civil engineering, Statistics

## Abstract

In the Longwall mining method, the coal face is being supported with two-legged or four-legged shield supports which improve better roof control between the face and canopy tip. The development of load on the face increases with the face retreating rate or the increase of the overhung length behind the shield support and as a result, the coal face tends to yield/fail. The yield/failure zone in the face extends depending on intensity of developed load due to the depth of mining, panel size, height of excavation and geometry of the overhung. The broken coal from the yielded face may fall on the shearer, armoured flexible conveyor (AFC), shield supports or may hit the deployed manpower in the face and causes the face stoppage which in turn results in to the loss of the project. It is therefore, a study is conducted to understand the face yield/failure behaviour considering various geo-mining conditions based on input parameters such as main roof thickness, overhung length, and material type and setting load. This paper develops a unique statistical model to predict the yield/failure zone in the longwall face. For this purpose, a total of fifty-four (54) three-dimensional finite element models in ANSYS software are developed and analyzed considering Drucker-Prager failure criterion.

## Introduction

Longwall mining method is popular for high production, safety and winning deep underground coal seams. This technology has the robust machinery such as shields to support the face, shearer to cut the coal from face and armoured flexible conveyor (AFC) to transport the cutted coal. Out of these machines, the shields are the only key machines which interact with roof and floor and ensures the safety to the longwall face, manpower and other face machines^1,2^.

The increase of load on the shield supports and the longwall face is higher compared to other mining methods since this method extracts wider face. As the coal is cutted from the face by shearer and face advances, the main roof overhung grows to a sufficient length behind the shield support. As a result, main roof may break in front of the longwall face. If the roof strata break ahead of the face, it causes heavy load on the shield and sometimes air blast may occur. This concept is called as uncontrollable periodic weighting. In case of weak and thin roof, the growth of main roof overhung or cantilever beam is short, however, for massive and thicker roof, this interval is very high. In case of hard, massive and thick main roof, overhung or cantilever may grow to a considerable length. Then main roof overhung and other roof rock layers will deflect and may separate with each other^3^. Due to this, the longwall face may yield and the broken pieces of coal from the face may hit AFC, shearer, shield support and manpower deployed in the face and causes the frequent stoppage of coal face. This stoppage of face retreating can lead to the high weighting and roof cavities. After the long exposure of such conditions, the shield stabilization may also happen if the face is not clear and face machinery are not advanced in time. Many studies reported that the development of load on the face and support depends on the depth of workings, height of excavation, width of face, main roof overhung length, its thickness and material type^4–10^. It is also reported that the face may yield if the load/stress on the face exceeds the strength of the longwall face.

Deb^11^ conducted the study on shield pressure data, the main roof can break about 3–6 m ahead of the face. This is particularly true if the main roof is weak and thick. Nemcik et al.^12^ investigated the longwall floor failure based on multiple sliding block model. Heasley et al.^13^ investigated the rock failure around the deep longwall panel using micro seismic events captured by the geophones. Kelly et al.^14^, also conducted a study to investigate the rock mass failure ahead of the face. In their study, the geophones were used to capture the seismic energy released by the rock when it fails. This study also reported that the rock mass breaks about 3–5 m ahead of the face. Bai et al.^15^ analyzed the brittle failure of coal wall in longwall face based on field investigation and numerical modelling. In their analysis, three coal wall types were considered to examine the wall spalling and found as 0.91 m, 0.6 m and 0.6 m for intact, vertical discontinuities and criss-cross discontinuities of coal wall respectively. Bai et al.^16^ investigated the coal wall spall in the longwall face using FLAC2D software and pointed out that the maximum vertical stress of 38.5 MPa is developed at 3.2 m in front of the face and then it declines very sharply at 1 m of the face. Also, the horizontal deformation of the wall was observed as a maximum when the face was at less than 2 m from the monitoring station. Aghababei et al.^4^ examined the longwall face floor failure using risk analysis for Parvedh-I coal mine. Prusek et al.^17^ investigated the roof fall risk in longwall mines using a novel algorithm combining the empirical methods and expert opinion and reported that a yield zone in the face and cavity in the roof of 2 m and 3 m respectively. Song et al.^18^ investigated the stability of longwall face using 2D finite element modelling technique and reported that maximum of 2 m yield zone occurs in the immediate roof and floor. Song and Chugh^19^ analyzed the stability of longwall face deployed in the thick seams and observed that the maximum yielding of 2 m in the face and also mentioned that the structural failure ahead of the longwall face depends on the seam height, properties of coal, immediate roof and floor, loading characteristics of gob and performance of powered supports. Kong et al.^20^ investigated the various parameters such as mining depth, mining height, cohesion of rock mass and support strength effecting the failure at the longwall face using 2D Universal Distinct Element Code software. Among these parameters, mining depth, mining height and cohesion showed significant influence on the face failure. Tien et al.^21^ conducted the study to determine deformation and failure of the coal wall at longwall face by developing physical model. Wojtecki et al.^22^ carried out the study for the influence zone of the failure in overlying and underlying rock from the seam under extraction using seismic hazards. Murmu and Budi^23^ investigated the longwall face failure during the main weighting period by estimating six parameters such as front abutment pressure, horizontal displacement, yielding condition, cohesion, plastic shear strain and plastic tensile strain, and reported that maximum yield zone of 5 m occurred.

From the above, it is understood that few studies were undertaken for estimation, analysis, and prediction of the yield zone of the face. Hence, it is imperative to develop a procedure to determine and predict the yield zone of the longwall face to extract the coal safely. Therefore, in this study, fifty-four (27 for setting load condition and 27 for web cutting condition) three-dimensional numerical models were prepared considering different variations in main roof overhung such as length (MRL), thickness (MRT) and material type (MRM). The yield zone data of all the numerical models were extracted and analysed to develop a novel index namely, yield zone index (YZI) to forecast the yield zone of the longwall face.

## Longwall mine site description

Adriyala Longwall Mine is located in the ALP area of the Singareni Collieries Company Limited, Telangana, India. The mine has three seams such as 1, 2 and 3 having an average thickness of 6.74 m, 6.1 m and 10 m respectively. The longwall technology is deployed in the seam 1 and two panels (1 and 2) were excavated successfully and panel 3 is under extraction. This paper is prepared based on the study conducted for panel 1. The depth of the study panel and mining height were 417 m and 3.2 m respectively. The shield supports of 1.71 m wide were deployed in the longwall face of 250 m width. The length of the panel was 2500 m. The maximum capacity of the shield support was 2 $\times$ 1152 T.

**Figure 1:**
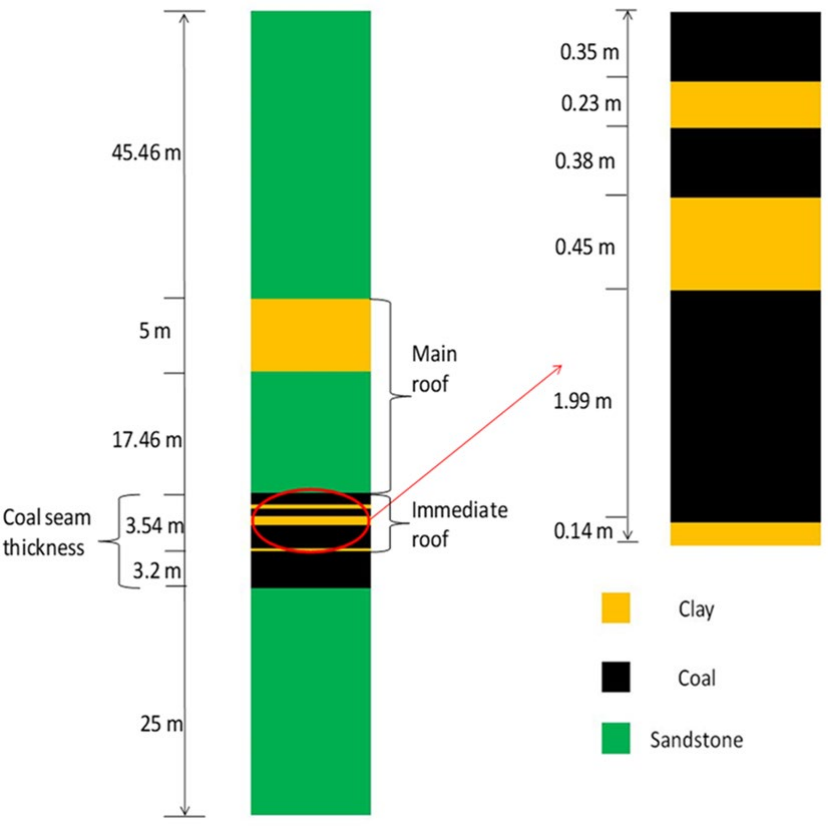
Borehole section of the panel.

Figure 1 shows the borehole section of the panel 1. From the thickness of seam 1 (6.74 m), bottom 3.2 m was excavated by the longwall and the rest thickness of 3.54 m was coal, clay and thin streaks of shaley coal considered as immediate roof. The roof rock strata lying above the immediate roof was sandstone (variable in thickness along panel length) considered as main roof and the rock layer below the coal seam/floor of the working was also sandstone. Therefore, the shield supports interact with three types of rock strata such as coal, clay and sandstone.

The first main fall in the panel 1of the Adriyala mine was reported at a retreat distance of 25 m from the setup room and the subsequent periodic falls were noticed between 10 and 15 m. It was also observed that the longwall face and roof yielded severely during the retreating and caused to engage the shearer in clearing the fallen coal in front of the AFC.

Physico-mechanical properties of the Adriyala mine site such as compressive strength $({\sigma }_{ci})$, modulus of elasticity $({E}_{ci})$ and geological strength index $(GSI)$ were collected and given in Table 1. It was noticed that the compressive strength and elastic modulus of sandstone (main roof) vary along the panel length as well as its thickness. As given in Table 1, ${m}_{i}$ is Hoek–Brown parameter and $D$ is disturbance factor was considered as 0 as the longwall method does not have any blasting effect^2^.

**Table 1:** Intact rock properties.

Rock strata	${\sigma }_{ci},\mathrm{MPa}$	$GSI$	${E}_{ci}, \mathrm{GPa}$	$D$	${m}_{i}$
Clay	17.5	50	4	0	7
Coal	28	50	5	0	7
Sandstone	35	60	9.87	0	10

The intact rock properties as mentioned in Table 1 were processed in RocLab software to estimate the rock mass properties of study panel. Table 2 shows the rock mass properties such as compressive strength $({\sigma }_{cm})$, modulus of elasticity $({E}_{cm})$, cohesion (${C}_{m}$), friction angle (${\phi }_{m}$), dilation angle (${\delta }_{m}$) based on^24^, density $(\rho )$ and poison’s ratio $(\nu )$.

**Table 2:** Rock mass material properties used in the study.

Rock layer	$\rho ,\mathrm{kg}/{\mathrm{m}}^{3}$	${\sigma }_{cm},\mathrm{MPa}$	${E}_{cm}, \mathrm{GPa}$	$\nu$	${C}_{m}, \mathrm{MPa}$	${{\phi }_{m },} \; {\circ}$	${{{\delta }_{m}},} \, {\circ}$
Clay	1100	2.582	1.278	0.35	0.811	27	18
Coal	1500	4.13	1.535	0.35	1.00	31	21
Sandstone	2147	7.643	5.132	0.28	1.461	38	19

After detailed investigation of the boreholes in the study panel, it was found that thickness of main roof overhung (MR) lies between 10 m and 22.5 m at an average of 15 m. Hence, in the study, MRT was considered as 10 m, 15 m and 22.5 m respectively as shown in Table 3. As mentioned earlier, the periodical falls were observed between 10 m and 20 m at an average of 13 m. Therefore, MRL was taken as 10 m, 13 m and 20 m.

**Table 3:** **V**ariations of the main roof (MR).

MR overhung length $(MRL)$, m	MR overhung thickness $(MRT)$, m	MR overhung material $(MRM)$
$MRL1=10$	$MRT1=10$	$MRM1$
$MRL2=13$	$MRT2=15$	$MRM2$
$MRL3=20$	$MRT3=22.5$	$MRM2$

Table 3 lists the variations of parameter of main roof overhung (MR) and Table 4 shows the MR overhung material type of Table 3.

**Table 4:** Material properties of MR overhung materials applied in the models.

MR overhung material	$\rho ,\mathrm{kg}/{\mathrm{m}}^{3}$	${\sigma }_{cm},\mathrm{MPa}$	${E}_{cm}, \mathrm{GPa}$	$\nu$	${C}_{m}, \mathrm{MPa}$	${{\phi }_{m },}$ °	${{{\delta }_{m}},}$ °
$MRM1$	2147	7.643	5.132	0.28	1.461	38	19
$MRM2$	2147	11.464	7.700	0.28	1.768	41	20
$MRM2$	2147	15.285	10.265	0.28	2.043	43	22

## Numerical model of longwall panel consists of shield support and other rock strata

**Figure 2:**
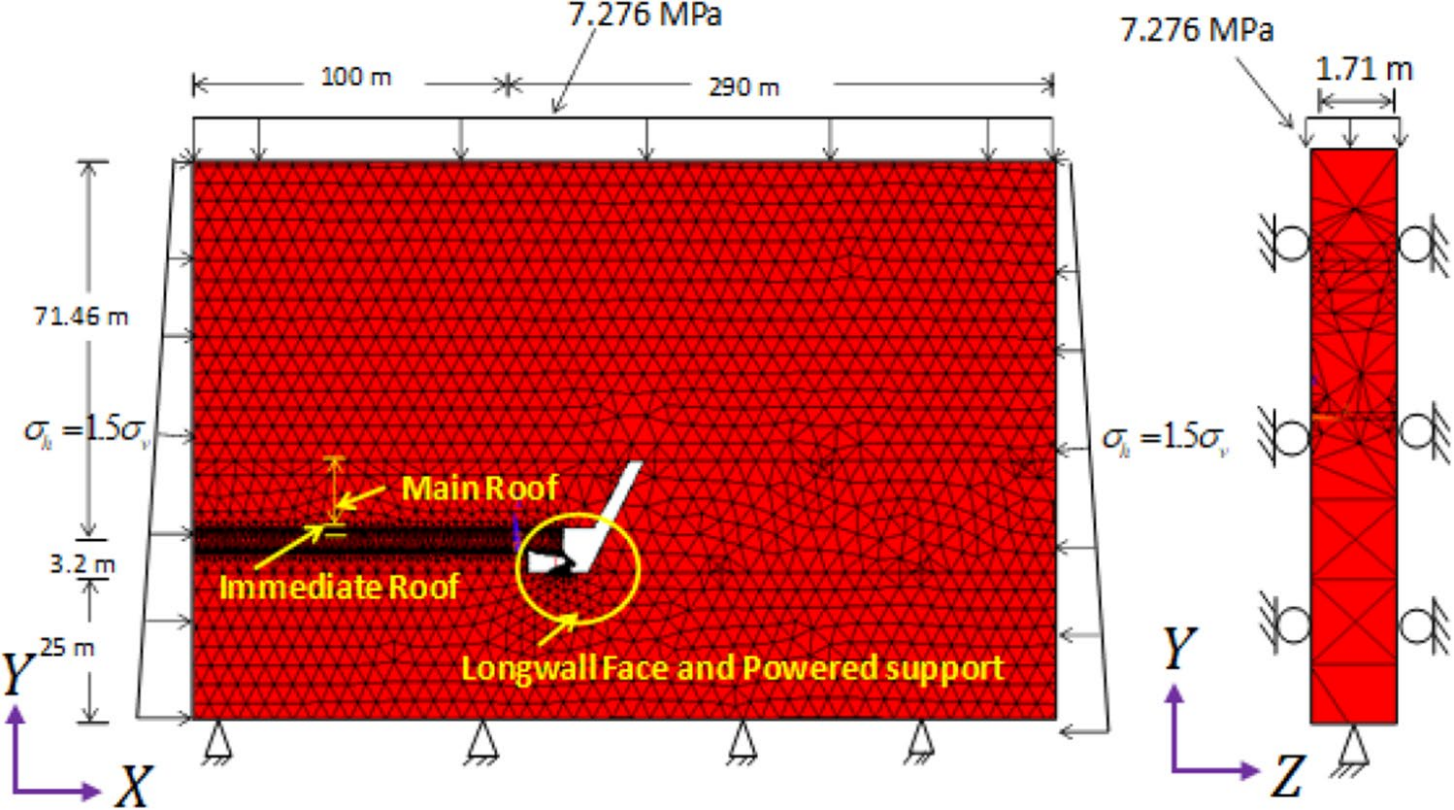
3D numerical model of longwall panel consisting of shield support, goaf and other coal bearing strata.

**Figure 3:**
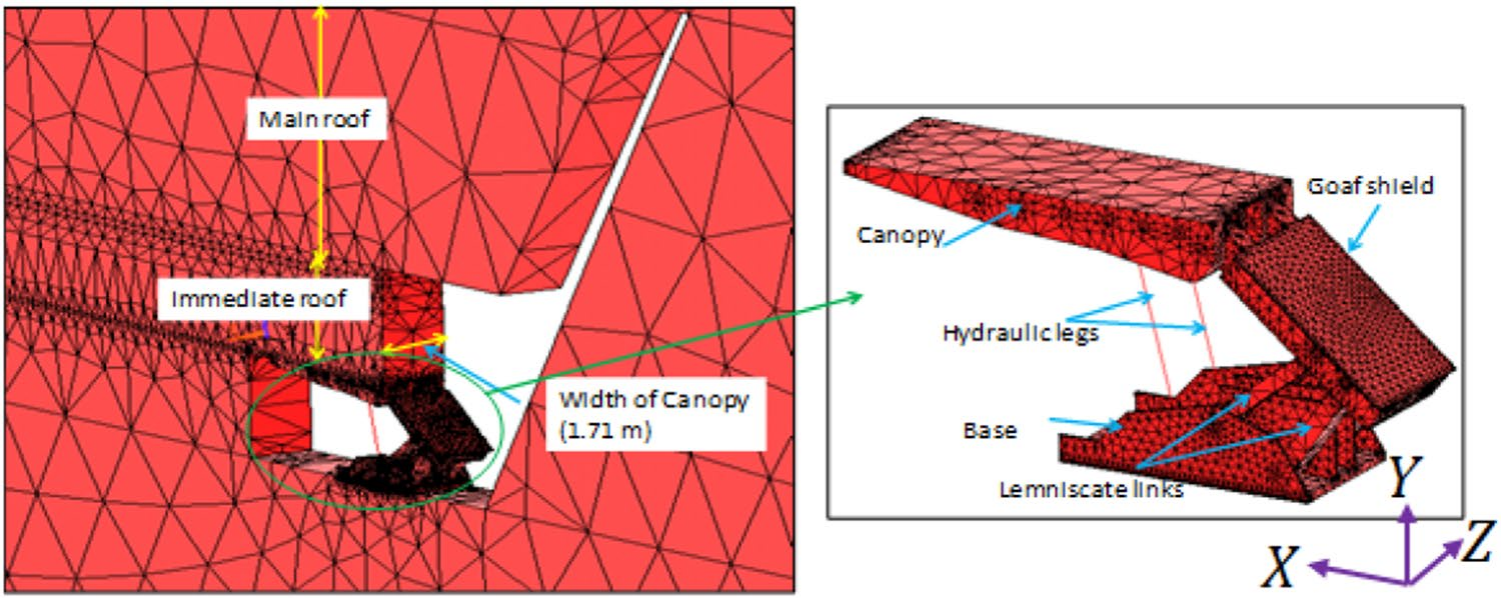
Zoomed view of 3D numerical model showing the shield, immediate roof and main roof.

A full-scale three-dimensional numerical model was prepared based on the borehole section of the study area (as mentioned in Fig. 1), panel data and engineering diagrams of the shield support. Each model consists of shield support, coal seam, immediate roof, main roof, coal bearing strata and goaf. The shield support of 2-legged 1152 T capacity was modelled and shield contains canopy, goaf shield, lemniscates links, base and hydraulic legs. The size of the numerical model was 1.71 m (equivalent to the width of the shield support) wide, 390 m length and 99.66 m height. The height and depth of mining were 3.2 m and 417 m respectively. Figures 2 and 3 show the development of 3D numerical model of the longwall panel. The clearance between the face and canopy tip was 0.1 m.

The thickness of rock layer below the floor and above the roof of working was considered as 25 m and 71.46 m respectively. The additional pressure of 7.276 MPa or 345.48 m overburden was applied on top of the model. As shown in Fig. 2, the faces perpendicular to X-axis and Z-axis were applied horizontal stress ${(\sigma }_{h}={1.5\times \sigma }_{h})$ and constrained in Z directions respectively. Bottom of the model was also constrained in Y-direction.

Fifty-four (54), 3D numerical models were developed considering the main roof overhung parameters such as MRL, MRT and MRM. Each parameter has three variations and these variations were done for both setting load and web cutting conditions. For simulating setting load models, the equivalent force of setting pressure ${(P}_{s})$ was 0.6 times of yield pressure (45 MPa) or 27 MPa was applied and estimated the setting strain. This strain was used for the development of multi-linear stress strain relationship and applied for hydraulic legs in web cutting models. All the condition of setting load models was kept same in web cut models, however, a web cut of 0.85 m was deleted and hydraulic legs in the form of bar element were inserted. All the numerical models were analysed based on the Drucker-Prager yield criterion. Detailed setting and wet cutting conditions were given in^25^.

## Results and discussions of the study

### Vertical stress distributions

**Figure 4:**
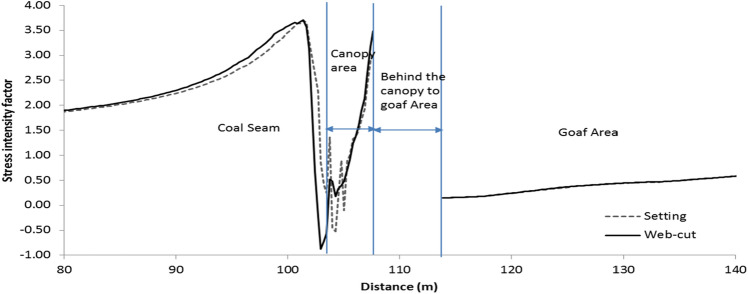
Vertical stress intensity factor profile in the longwall panel.

Vertical stress intensity factor was defined as the ratio between induced stress and insitu stress during the retreating of face and its profile was estimated just above the shield support/roof along the panel length. Figure 4 shows the stress intensity factor for setting load (dotted line) and web-cutting conditions (dark line). From this Figure, it was observed that high tensile stress develops at the tip of the canopy and compressive stress develops on the rear side of the canopy. As expected, that high stress developed in the web cutting models due to cutting of 0.85 m web from the face.

### Distribution of load on the shield canopy

**Figure 5:**
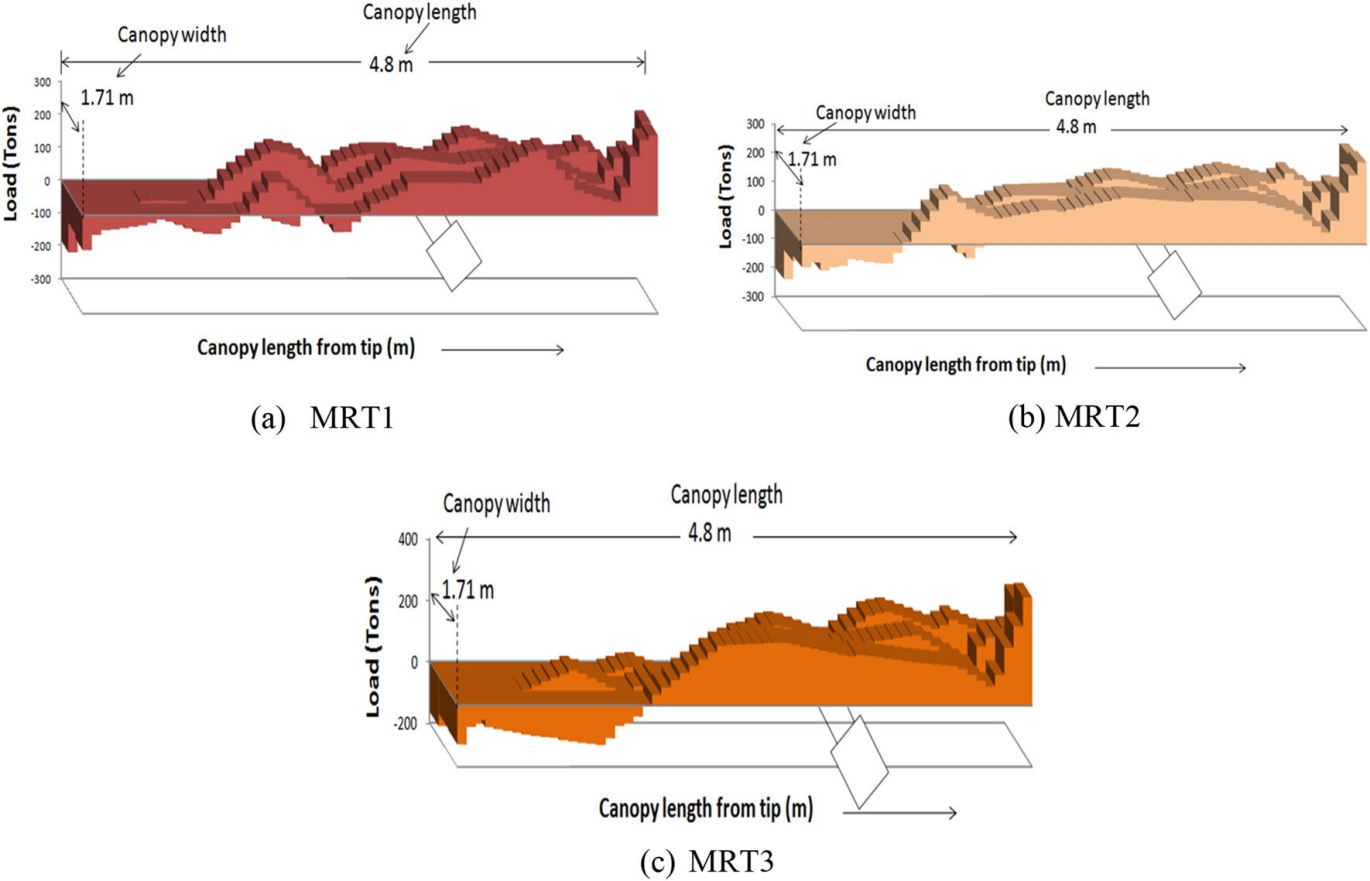
Distribution of load on the canopy of MRL1M1(T1 to T3) models.

Figure 5 shows the typical load distribution on top of the canopy for web cut models of MRL1M1 (T1–T3). From the figure, it is clear that tensile load develops at the tip of the canopy and the compressive load develops towards rear side of the canopy. Also, the development of load on the canopy increases with the increase of main roof thickness as 242, 285 and 324 tonnes for 10, 15 and 22.5 m respectively.

### Roof convergence

**Figure 6:**
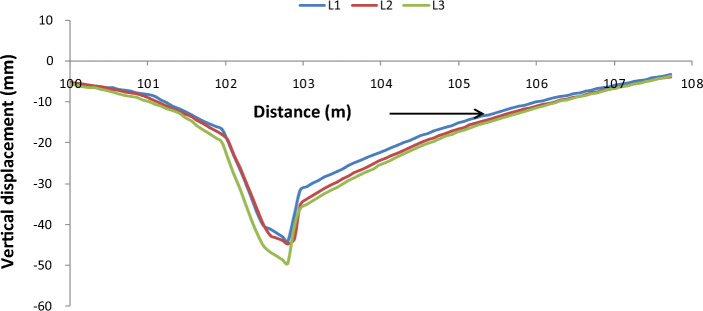
Roof convergence distribution for variation of main roof overhung length.

The roof convergence in the longwall face was obtained after subtracting the vertical displacement of the setting models from the that of web cutting models^26^. The roof convergence profiles are plotted for MRT2M2 (L1–L3) models by varying the main roof overhung lengths MRL1 = 10 m, MRL2 = 13 m and MRL3 = 20 m and they are shown in Fig. 6.

A maximum vertical displacement of 42.72 mm, 43.62 mm and 48.7 mm occurred due to web cutting for main roof overhung lengths MRL1, MRL2 and MRL3 respectively. It is clearly shown that the vertical displacement on top of the coal seam near the longwall face increases with an increase of main roof overhung length from MRL1 or 10 m to MRL3 or 20 m.

### Deterioration of the longwall face area

**Figure 7:**
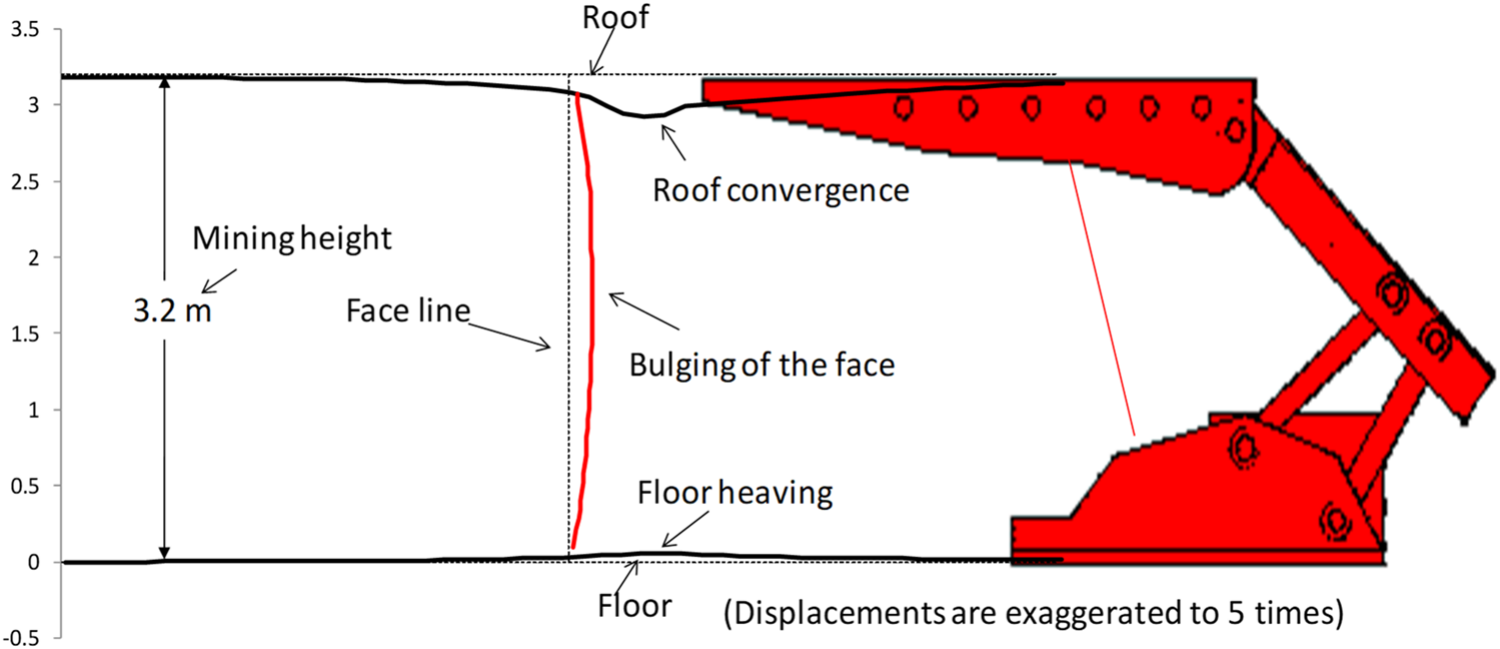
Bulging of the longwall face.

Vertical displacement distributions around the shield and longwall face area are shown in Fig. 6. It is clear that the coal face is yielded and low stress is developed near the face area. This implies that dilation of coal face is occurring and is evident from the vertical as well as the horizontal displacements as shown in Fig. 7. This Figure shows that the coal face bulges towards the shield. This phenomenon was observed in all the models having different amount of dilation depends on main roof overhung length (MRL), overhung thickness (MRT) and overhung material type (MRM).

## Methodology for estimation of the yield zone in the longwall face

**Figure 8:**
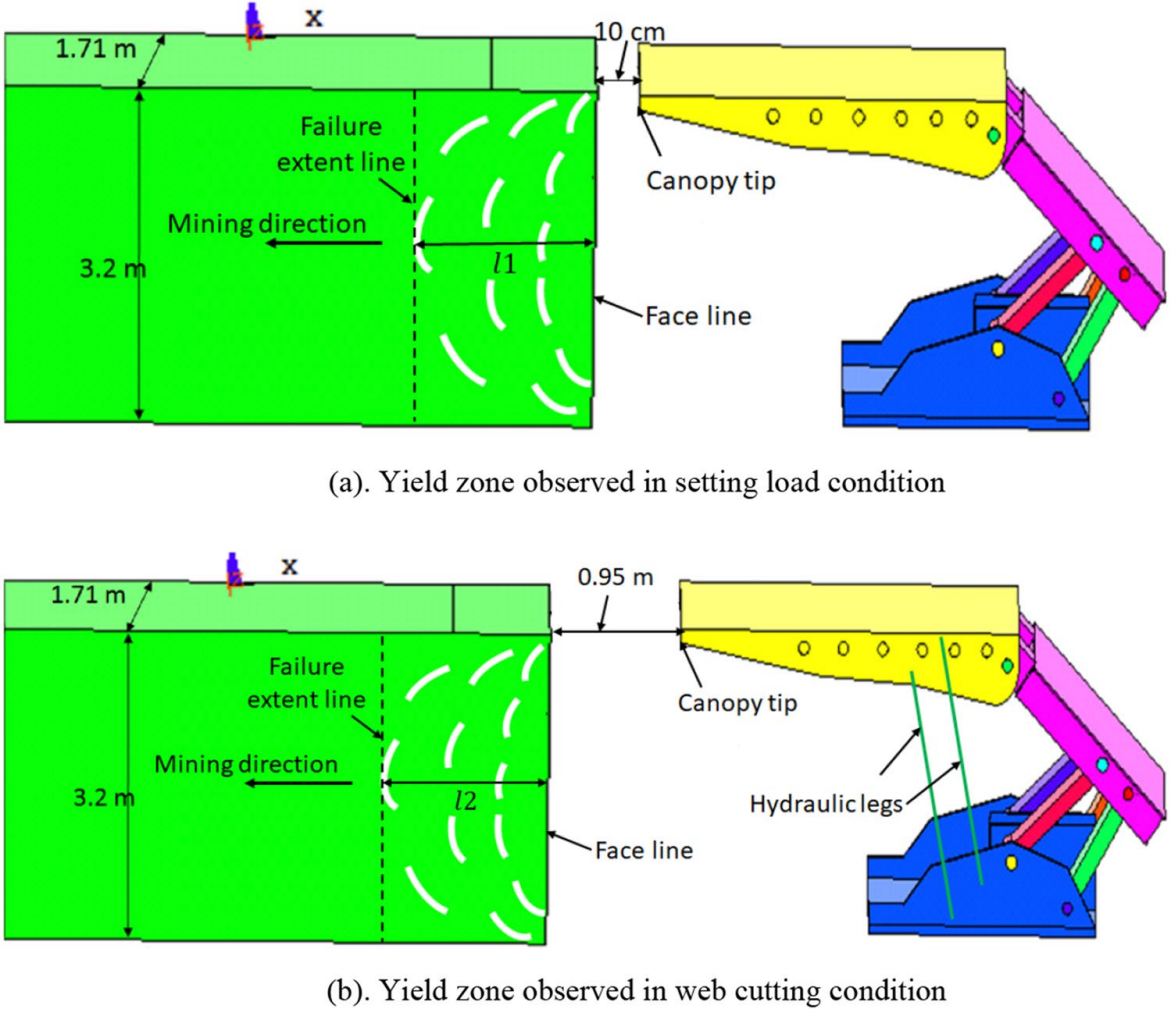
Determination of yield zone in setting load and web cutting models.

As mentioned above, clearance between the tip and the longwall face was kept as 10 cm in all the finite element models. After cutting of the web cut of 0.85 m, the tip to face clearance became 0.95 m. For estimation of the yield zone in the longwall face, the maximum plastic intensity of 0.01 was kept for all the numerical models. Then, the length of the yield zone from the face line to inside the longwall face was measured as ‘$l1$’ for setting load models as shown in Fig. 8a. Similarly, for web cutting models, the yield zone length was measured from the face line as ‘$l2$’ (Fig. 8b). The yield zone observed in the setting load models ($l1$) was found as almost the same as the yield zone of the web cutting models ($l2$). Hence, the yield zone obtained in setting load models was not considered in further analysis.

### Development of yield zone in the longwall panel

In the study, longwall face, the layers of immediate roof and a part of main roof (for a few cases) were observed as yielded. The study finds that the maximum failure zone occurs in the coal seam and extends inside the face. From the investigation, it was found that the face and immediate roof yields severely in in all the models. The minimum and maximum yield zones were observed as 1.5 m and 3.0 m respectively. This similar phenomenon was also observed in the mine and discussed in detailed in the below section.

**Figure 9:**
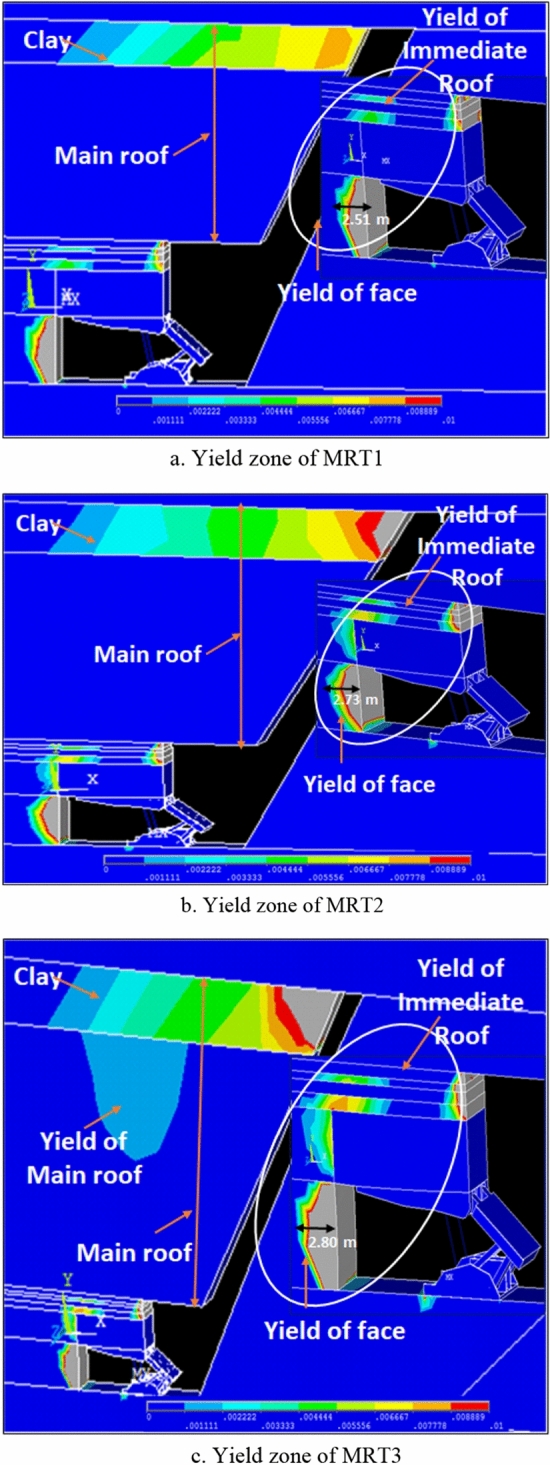
Development of yield zone for various combination of main roof.

Yield zone was estimated based on the equivalent effective plastic strain developed in the coal face and other part of the roof strata. It may be noted that higher value of effective plastic strain indicates the severe yielding. In Fig. 9a–c, yield zone around the face area is shown for variation of main roof thickness from MRT1, MRT2 and MRT3 respectively. From these figures, it was observed that the development of plastic strain intensity enhances with the growing of the main roof thickness. The yield zone of 2.51 m, 2.73 m and 2.8 m occurred for MRT1 = 10 m, MRT2 = 15 m and MRT3 = 22.5 m respectively. It can be observed that growth of 10% yield zone occurred with the increment of MRT from 10 to 22.5 m. For MRT3 model, the severity of yield zone increased and it can be found in the main roof also. The development of yield zone severity was observed less in other two combinations of the models.

It is clear that if main roof thickness is 22.5 m or T3 models, it yields irrespective of variation in other parameters. Another significant observation was that immediate roof mostly yields in all models. The part of coal seam layer exists above the main roof was also yielded in all models as shown in the Fig. 9.

#### Effect of the main roof overhung length

**Figure 10:**
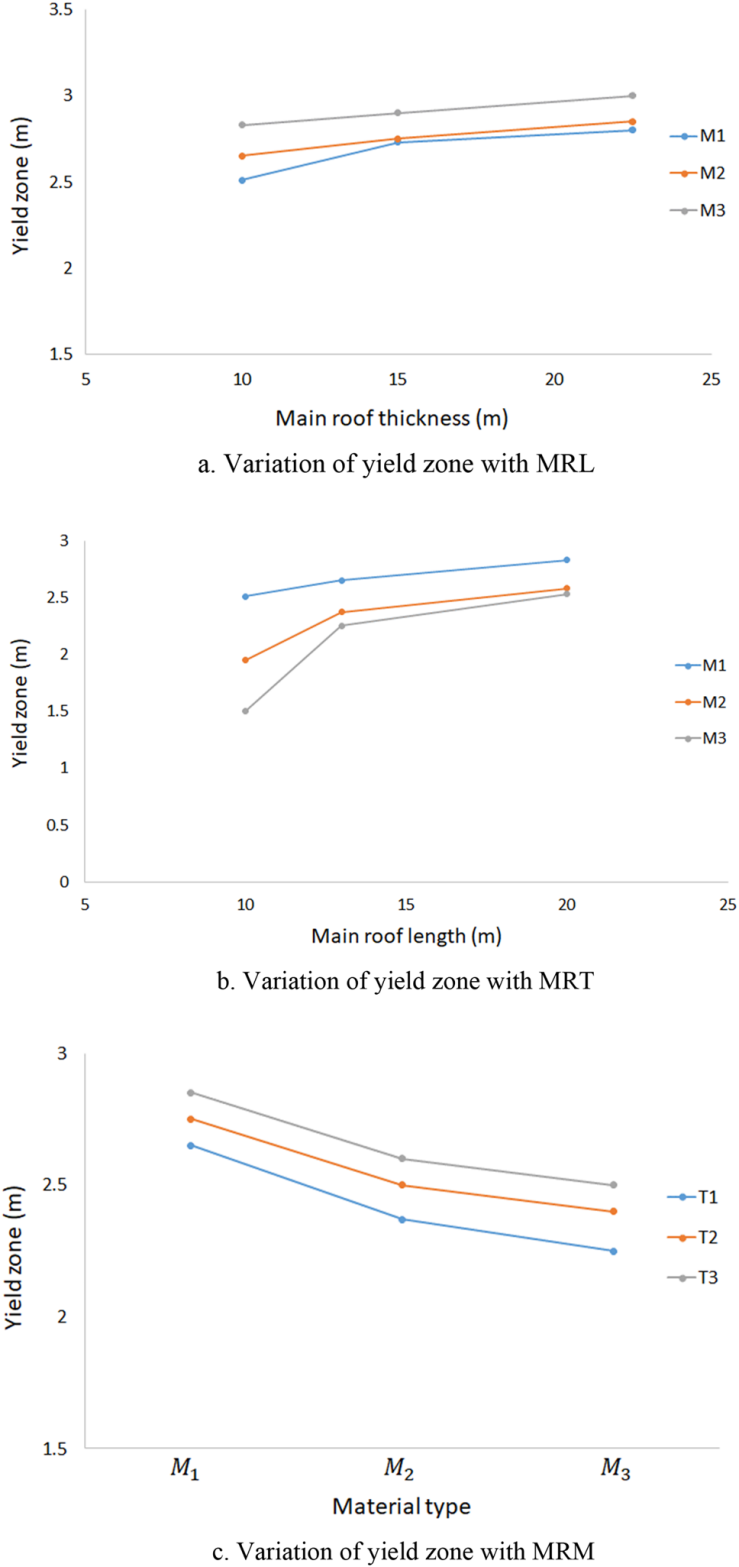
Behaviour of yield zone with different main roof parameters.

It is observed that the load on the face and shield support increases with the increment of the main roof overhung, as a result, the failure in the longwall face can be noticed and yield zone increases with the increment of main roof overhung length. It is found that growth of 11% yield zone occurred with the increment of MRL from 10 to 20 m. The yield zone of 2.51 m, 2.65 m and 2.83 m was observed for the main roof overhung length of 10 m, 13 m and 20 m respectively as shown in Fig. 10a.

#### Effect of the main roof overhung thickness

As mentioned in many literatures, the formation of thicker roof behind the shield support/longwall face enhances the load on the face and causes the increment of yield zone in the longwall face. In this study also, the same phenomenon was observed (Fig. 10b). For a main roof overhung thickness of 10 m, 15 m and 22.5 m, the yield zone of 2.83 m, 2.90 m and 3.0 m was observed respectively.

#### Effect of the main roof material type

The stiffer/strong material causes a low deformation and develops low-stress concentration on the workings than the weaker/softer rock strata. As a result, the low failure/yield zone is observed in the longwall face. From the Fig. 10c, it is also proven that the more yield zone occurred in the M1 (or softer) models, and low yield zone occurred in M3 (or strong) models. The yield zone of 2.85 m, 2.60 m and 2.50 m occurred for M1(softer), M2 (medium) and M3 (strong) models respectively.

It can be clear that in all the combinations, the immediate roof develops yield zone and may tend the cavity formation in case of L3T3 combinations of the models. This may result into falling of the roof rock pieces and face slabbing.

## Validation of the yield zone observed in the numerical analysis

In this section the yield zone observed in the longwall face from all the numerical models is validated with the field observed values and the earlier published literature.

**Figure 11:**
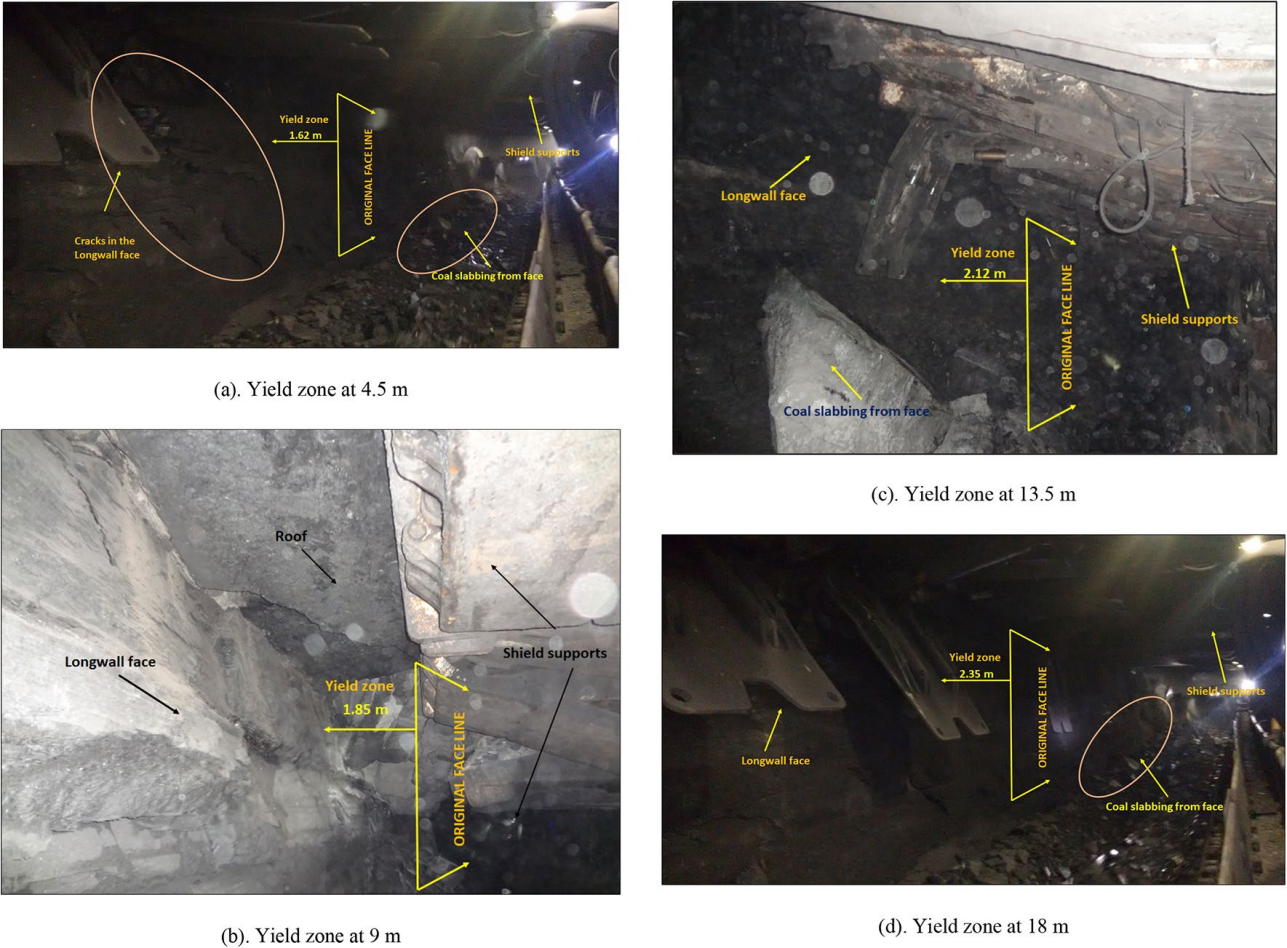
Yield zone of the longwall face observed in the study mine.

As mentioned in “Longwall mine site description” section, the average periodic weighting interval was observed between 12 and 20 m. After occurrence of periodic fall, the load on the shield support and the face was observed minimum due to occurrence of the main fall. Few field measurements of the yield zone were taken in the face with an extensometer at 4.5 m, 9.0 m, 13.5 m and 18 m face advance after a periodic fall. The yield zone of 1.62 m, 1.85 m, 2.12 m and 2.35 m were observed at face retreat of 4.5 m, 9.0 m, 13.5 m and 18 m respectively. Figure 11a–d shows the yield zones of the longwall face at advance of 4.5 m, 9.0 m, 13.5 m and 18 m respectively. From these Figures, it can also be observed that most of the coal is slabbing, roof is yielding and boulders are fallen from the face. Due to this phenomenon, many time the AFC and shearer were engaged for cleaning the coal which resulted for delay in production, increase of the roof weighting and a reduction of periodic weighting interval.

This measured yield zone in the face is almost correlating with that of the numerical modelling results.

**Figure 12:**
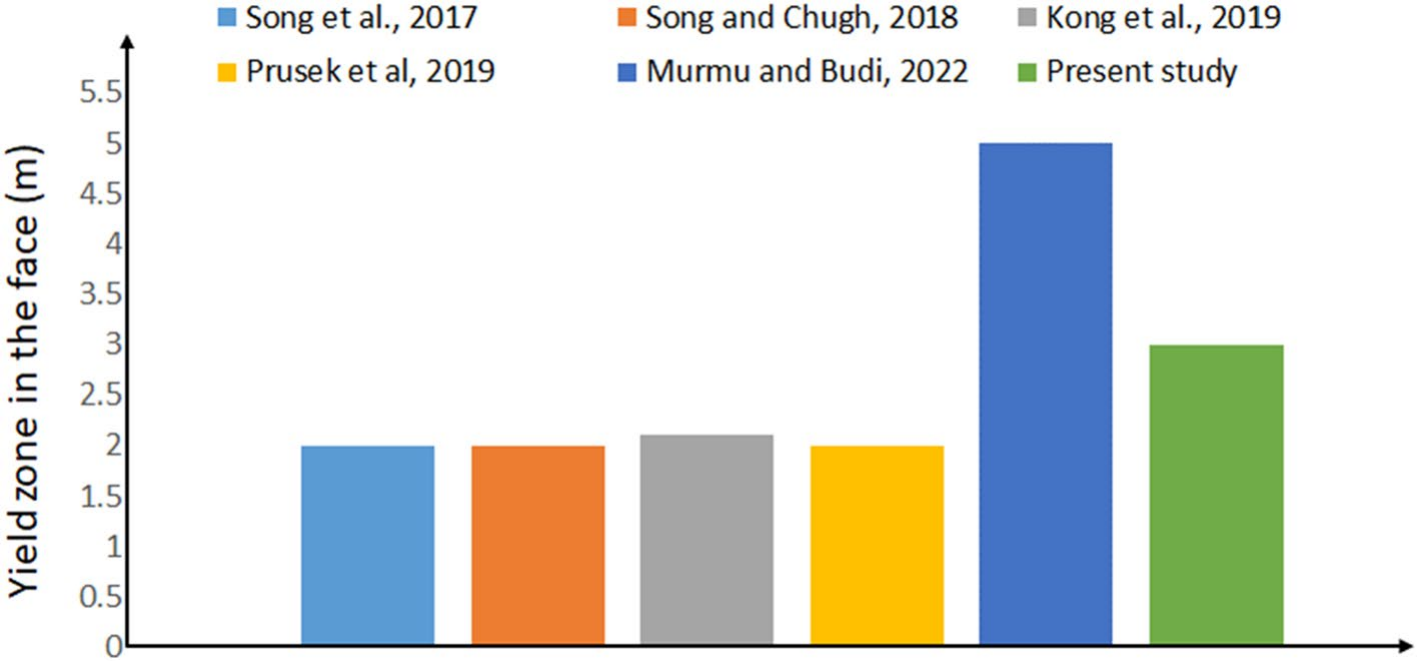
Validation of the yield zone observed in this study with literature.

Very few studies^17–20,23^ were undertaken to estimate the yield zone in the longwall face, floor and immediate roof. Figure 12 shows the validation of the yield zone observed in this study with the published literature. From this Figure, it can be understood that the maximum of 5 m yield zone occurred in^23^ and that of 2 m minimum occurred in^18^. It is also reported that the 79.4% of face falls extended less than 1 m, 20% lies between 1 and 2 m, 0.6% lies beyond 2 m at longwall face (Face # 1306, at a depth of 637 m) of Zhaozhuang coal mine in China. Kong et al.^20^ reported that a maximum of 2.1 m face failure occurs and also mentioned that face failure extension varies proportional to cohesion of coal and support strength and inversely proportion to mining depth and mining height. It is found that an average of 2 m yield zone occurred in all other studies. However, in the present study develops the maximum yield zone of 3 m which is almost matches with them.

## Development of yield zone index for prediction of the yield zone in the longwall face

**Figure 13:**
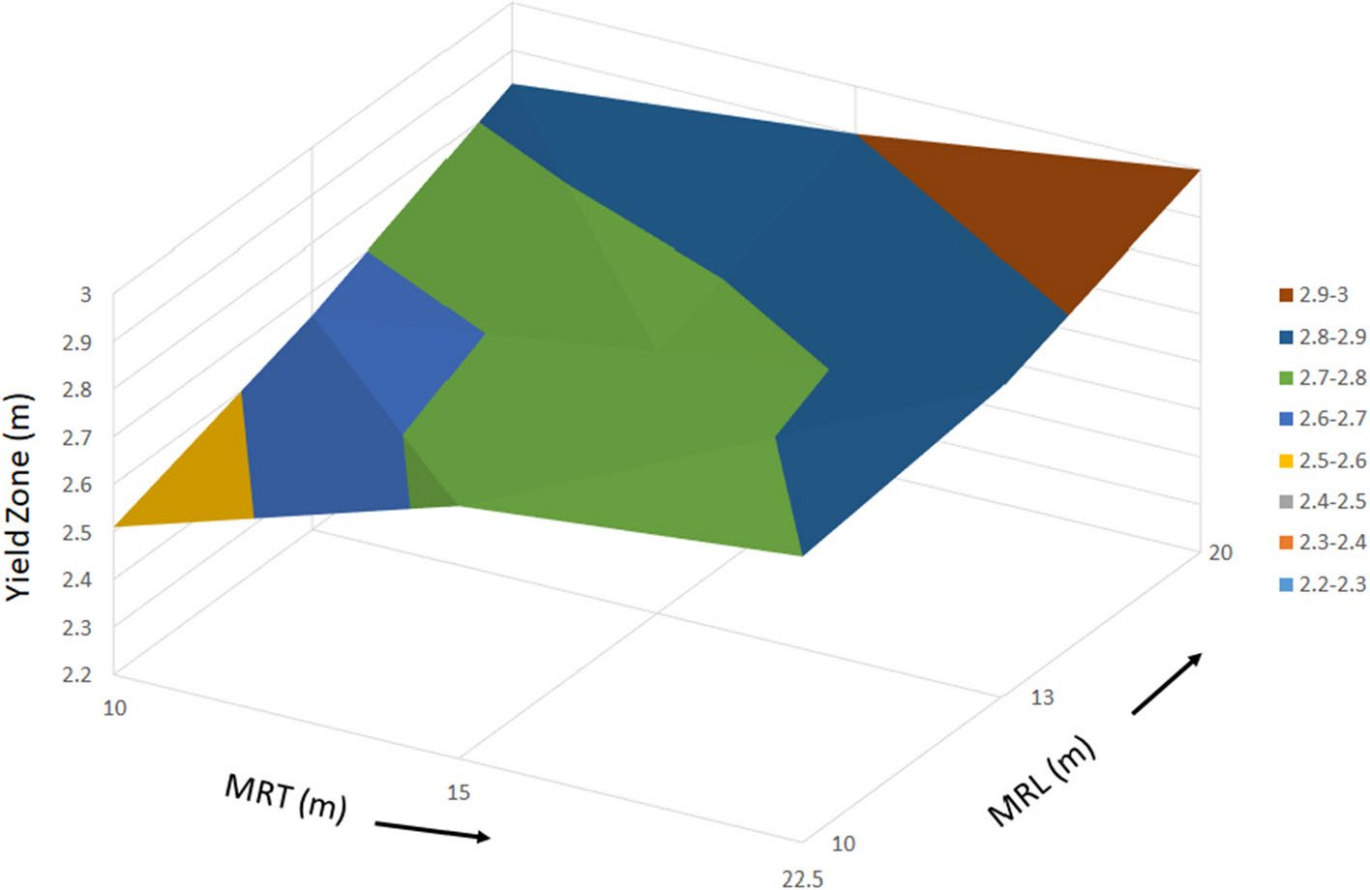
Surface plot of yield zone for M1 material type.

As shown in the Table 5, a total of 54, 3D finite element models were developed to estimate the occurrence of yield zone in the longwall face by varying the main roof parameters such as its length, thickness and material properties. As shown in the Fig. 10a–c, the occurrence of yield zone can be noticed as an increasing trend with the growth of longer and thicker main roof and decreasing trend with the increment of material properties (from softer to strong). The variations of the yield zone with the variable parameters observed in a linear trend. As mentioned above, the yield zone in the setting load model and web cutting model was found almost same. Hence, the yield zone values (27 observations) of setting load models were not considered. Table 5 shows the yield zone values of web cutting models (27 observations) and considered in the analysis. Also, for the development of the statistical model for yield zone index, the elastic modulus of the main roof was taken to consider the effect of the material type. Figure 13 shows the 3D surface plot of yield zone occurred in the longwall face for M1 material type.

**Table 5:** Yield zone of the longwall face observed in the study.

MRL (m)	MRT (m)	MRM (GPa)	Yield zone(m)	Whether main roof yielded
MRL1 = 10	MRT1 = 10	MRM1 = 5.132	2.51	No
	MRT2 = 15		2.73	No
	MRT3 = 22.5		2.8	Yes
MRL2 = 13	MRT1 = 10		2.65	No
	MRT2 = 15		2.75	No
	MRT3 = 22.5		2.85	Yes
MRL3 = 20	MRT1 = 10		2.83	No
	MRT2 = 15		2.9	No
	MRT3 = 22.5		3	Yes
MRL1 = 10	MRT1 = 10	MRM2 = 7.7	1.95	No
	MRT2 = 15		2.05	No
	MRT3 = 22.5		2.3	Yes
MRL2 = 13	MRT1 = 10		2.37	No
	MRT2 = 15		2.5	No
	MRT3 = 22.5		2.6	Yes
MRL3 = 20	MRT1 = 10		2.58	No
	MRT2 = 15		2.65	No
	MRT3 = 22.5		2.7	Yes
MRL1 = 10	MRT1 = 10	MRM3 = 10.265	1.5	No
	MRT2 = 15		1.7	No
	MRT3 = 22.5		1.73	Yes
MRL2 = 13	MRT1 = 10		2.25	No
	MRT2 = 15		2.4	No
	MRT3 = 22.5		2.5	Yes
MRL3 = 20	MRT1 = 10		2.53	No
	MRT2 = 15		2.58	No
	MRT3 = 22.5		2.65	Yes

Hence, in order to combine the effect of all the input parameters *(MRL, MRT and MRM),* Yield Zone Index (YZI) was developed using statistical analysis to predict the yield zone for similar longwall mining condition.

In this study, YZI is the dependent variable, and input parameters such as main roof overhung length, thickness and material type are independent variables. From the statistical analysis, the best fit statistical model was developed to determine the condition of the longwall face by estimating yield zone in the longwall panel. The coefficient of determination for the model was 0.77. The yield zone index of the longwall panel was developed as given in Eq. (1).

1 $$YZI={p}_{0}+{p}_{1}\times MRL+{p}_{2}\times MRT-{p}_{3}\times MRM$$

The coefficients of the above statistical model were estimated by performing the multiple regression analysis as ${p}_{0}$, ${p}_{1}$, ${p}_{2}$ and ${p}_{3}$ are evaluated to be 2.321, 0.051, 0.017 and -0.112 respectively. Hence, equation of $YZI$ is given below.

2 $$YZI=2.321+0.051\times MRL+0.017\times MRT-0.112\times MRM$$

where,$YZI$ is a yield zone index, $MRL$ is the main roof length (m), $MRT$ is main roof thickness (m), and $MRM$ is main roof elastic modulus.

## Conclusion

This paper proposes the methodology to estimate the yield zone of longwall face using 3D numerical models and develops the novel index “yield zone index (YZI)” by combining the effect of the main roof overhung (length, thickness and material type) to predict the condition of the longwall face before taking up the actual operation in similar geo-mining conditions.

From this study, it was observed that the yielding of the longwall face reduces as the main roof overhung becomes more competent from soft (MRM1) to strong (MRM3) and increases with increase of main roof overhung length from MRL1 to MRL3 and main roof thickness MRT1 to MRT3. The setting pressure did not show any effect on the phenomena of yielding of longwall face.

This study reveals that the maximum yield zone of 3 m occurs in the L3T3M1 model and the minimum yield zone of 1.5 m occurs in the L1T1M3 model. The results of the numerical models were also in agreement with field measured data.

## Data Availability

All data generated or analysed during this study are included in this published article.
